# Cytokine Levels in Human Vitreous in Proliferative Diabetic Retinopathy

**DOI:** 10.3390/cells10051069

**Published:** 2021-04-30

**Authors:** Dean F. Loporchio, Emily K. Tam, Jane Cho, Jaeyoon Chung, Gyungah R. Jun, Weiming Xia, Marissa G. Fiorello, Nicole H. Siegel, Steven Ness, Thor D. Stein, Manju L. Subramanian

**Affiliations:** 1 Department of Ophthalmology, Boston University School of Medicine & Boston Medical Center, Boston, MA 02118, USA; dean.loporchio@bmc.org (D.F.L.); kayi.tam@bmc.org (E.K.T.); janechomd@gmail.com (J.C.); marissa.fiorello@bmc.org (M.G.F.); nicole.siegel@bmc.org (N.H.S.); steven.ness@bmc.org (S.N.); 2 Department of Medicine (Biomedical Genetics), Boston University School of Medicine, 72 East Concord Street, Boston, MA 02118, USA; jychung@bu.edu (J.C.); gyungah@bu.edu (G.R.J.); 3 Department of Pharmacology and Experimental Therapeutics, Boston University School of Medicine, Boston, MA 02118, USA; wxia@bu.edu; 4 Geriatric Research Education and Clinical Center, VA Bedford Healthcare System, Bedford, MA 01730, USA; 5 Laboratory Medicine, Department of Pathology, Boston University School of Medicine, Boston, MA 02118, USA; tdstein@bu.edu; 6 VA Bedford Healthcare System, Bedford, MA 01730, USA; 7 VA Boston Healthcare System, Boston, MA 02130, USA

**Keywords:** diabetic retinopathy, vitreous cytokines, quantitative immunoassay, neuroinflammatory markers, vascular endothelial growth factor, interleukin-8, interleukin-15, interleukin-16, basic fibroblast growth factor

## Abstract

In this study, we compare the vitreous cytokine profile in patients with proliferative diabetic retinopathy (PDR) to that of patients without PDR. The identification of novel cytokines involved in the pathogenesis of PDR provides candidate therapeutic targets that may stand alone or work synergistically with current therapies in the management of diabetic retinopathy. Undiluted vitreous humor specimens were collected from 74 patients undergoing vitrectomy for various vitreoretinal disorders. Quantitative immunoassay was performed for a panel of 36 neuroinflammatory cytokines in each specimen and assessed to identify differences between PDR (*n* = 35) and non-PDR (*n* = 39) patients. Levels of interleukin-8 (IL-8), IL-15, IL-16, vascular endothelial growth factor (VEGF), VEGF-D, c-reactive protein (CRP), serum amyloid-A (SAA), and intracellular adhesion molecule-1 (ICAM1) were significantly increased in the vitreous of PDR patients compared to non-PDR patients (*p* < 0.05). We report novel increases in IL-15 and IL-16, in addition to the expected VEGF, in the human vitreous humor of patients with PDR. Additionally, we confirm the elevation of ICAM-1, VCAM-1, SAA, IL-8 and CRP in the vitreous of patients with PDR, which has previously been described.

## 1. Introduction

Diabetic retinopathy (DR) is the leading cause of visual impairment among working age adults in the United States (US) [1]. While DR currently impacts approximately one in three US diabetic adults over the age of 40 [2], the growing incidence of type 2 diabetes, fueled in part by the surge in obesity, will likely translate into an even greater prevalence of DR in the future [3].

Proliferative diabetic retinopathy (PDR), which is the most advanced stage of diabetic retinopathy, can be associated with severe visual impairment and blindness. It is characterized by retinal ischemia and an ischemia-induced upregulation of angiogenic factors, namely, vascular endothelial growth factor (VEGF) [1,4]. In recent years, anti-VEGF agents such as bevacizumab (Avastin, Genentech, San Francisco, CA, USA), ranibizumab (Lucentis, Genentech, San Francisco, CA, USA), and aflibercept (Eylea, Regeneron, Tarrytown, NY, USA) have played a prominent role in the treatment of diabetic retinopathy by reducing both the vascular leakage in diabetic macular edema (DME) and the angiogenesis in proliferative disease [1].

Current anti-VEGF medications are limited by their short duration of action, with patients requiring visits to their doctor and therapeutic intravitreal injections as frequently as once a month [5,6]. Additionally, multiple studies have demonstrated that along with pro-angiogenic factors, patients with PDR have elevated intraocular concentrations of inflammatory cytokines [6,7,8], raising the possibility that additional therapeutic avenues exist, beyond targeting VEGF, for the treatment of PDR. This study aims to characterize the vitreous cytokine profile in patients with PDR and compare it to the cytokine profiles of patients without PDR.

## 2. Materials and Methods

### 2.1. Inclusion Criteria

All patients 18 years or older with a primary language of English or Spanish scheduled for pars plana vitrectomy in at least one eye for a vitreoretinal disease, were included in this study (Institutional Review Board (or Ethics Committee) of Boston Medical Center and Boston University Medical Campus Institutional Review Board (Protocol Number H-33883, Date of Approval: 5/19/15). Patients with PDR, diabetic patients with mild, moderate and severe non-proliferative diabetic retinopathy, and those without a diagnosis of diabetes were included in this study. Surgical indications for patients in the PDR group included vitreous hemorrhage, tractional retinal detachment, and epiretinal membranes. Those without PDR included patients with epiretinal membranes (ERM), macular holes (MH), hemorrhagic posterior vitreous detachments (PVD), rhegmatogenous retinal detachments (RRD), lens subluxations, and vitreomacular traction (Table 1).

### 2.2. Biospecimen Collection

Vitreous samples were collected at the start of each vitrectomy procedure by the surgical team, and 0.5–1.0 mL of undiluted vitreous fluid was aspirated via the vitrectomy probe into an attached syringe. Saline was then infused into the vitreous cavity in order to repressurize the eye. The syringe containing the specimen was then capped using a sterile technique and directly handed to a research assistant who labelled it with a predetermined nonidentifiable study number and placed the sample on ice for transport. In the Molecular Genetics Core Laboratory (MGCL) at Boston University, the vitreous samples were aliquoted into 100 μL Eppendorf tubes and stored at −80 °C until analysis. Aside from collection of the vitreous samples, each study participant’s surgery was completed according to the clinical standard of care for that patient’s ocular condition.

### 2.3. Biospecimen Analyses

Vitreous samples were centrifuged for 15 min at 12,000 rpm to separate the cellular contents, and then aliquoted into eight 100 μL polypropylene tubes prior to freezing at −80 °C. Any remaining volume for each vitreous specimen was retained and stored at −80 °C. Quantitative immunoassay was performed using the Meso Scale Discovery (MSD, Rockville, MD, USA) system for a panel of 36 neuroinflammatory factors (Neuroinflammation Panel 1, K15210G, Meso Scale Discovery). Samples were analyzed for Proinflammatory panel 1 (interferon-gamma [IFN-γ], interleukin (IL) -1A, IL-10, IL-12p70, IL-13, IL-1β, IL-2, IL-4, IL-6, IL-8, tumor necrosis factor-alpha [TNF-α]), Cytokine panel 1 (IL-12/IL-23p40, IL-15, IL-16, IL-17A, IL-1α, IL-5, IL-7, TNF-β, VEGF-A), Chemokine panel 1 (monocyte chemoattractant protein-1 [MCP-1], MCP-4, Eotaxin, interferon-gamma inducible protein-10 [IP-10], Eotaxin-3, thymus and activation regulated chemokine [TARC], macrophage inflammatory protein-1 alpha [MIP-1α], MIP-1β), and Angiogenesis panel 1 (bFGF, VEGF receptor-1/Fms-like tyrosine kinase-1 [VEGFR-1/Flt-1], VEGF, Tie-2, VEGF-C, VEGF-D), Vascular injury panel 2 (Serum Amyloid A Protein [SAA], C-Reactive Protein [CRP], Vascular Cell Adhesion Molecule 1[VCAM-1], Intercellular Adhesion Molecule 1 [ICAM-1]) per the manufacturer’s instructions and run in duplicate. Sulfo-tag conjugated secondary antibody was used for signal detection, and an MSD SECTOR S 600 Imager was used to measure analyte levels (pg/mL).

### 2.4. Statistical Analyses

Our primary analysis examined the cytokine levels of patients with PDR (case group) and those without PDR (control group, which included patients without diabetes and diabetic patients with retinopathy that was less than proliferative). To identify differences in inflammatory marker levels, we conducted linear regression analysis for quantitative vitreous measures as the outcome and the disease status (PDR vs. no PDR) as the predictor, adjusting for sex and age as covariates using the R software. We did not adjust for race as a covariate due to the diverse nature of our cohort, that included five different race categories and individuals of mixed racial ancestry (almost 30% of our cohort identified as a race other than Black or White, and 14% identified as “other”), and a significant imbalance of racial breakdown in the case and control groups. A secondary multivariate linear regression analysis was conducted, adjusting for nonproliferative diabetic retinopathy, anti-VEGF treatment, and rhegmatogenous retinal detachments (RRD) as additional covariates. The measures of vitreous specimens were log-transformed to obtain a normal distribution. We calculated effect size for each cytokine to measure the strength of the relationship between disease state (PDR) and cytokine levels, for both the primary and secondary analyses; the change in effect size and p-value illustrates the impact of adjusting for the additional variables. False discovery rate (FDR) was applied in order to determine the rate of potential false positives in our study due to multiple comparisons with several outcomes, with an a priori cutoff at 0.10 (or 10%). In addition, we provide fold changes, which shows the ratios of mean levels between cases and controls. Finally, for the secondary analysis, we computed variance inflation factor (VIF) for assessing multicollinearity using the R library HH (https://cran.r-project.org/web/packages/HH/index.html, accessed on 13 April 2021) and evaluated model fit (i.e., goodness-of-fit) to the observed data using R^2^. We considered a value of VIF greater than 10 as an indication of inconsequential collinearity [9].

## 3. Results

There were 95 patients initially enrolled in the study. Of these, 15 were dropped for various reasons, including inability to obtain the desired specimen/inadequate sample collection (*n* = 5), loss to follow-up (*n* = 3), withdrawal of consent on the day of surgery (*n* = 1), surgery cancellation (*n* = 4), and mismatch of sample identification numbers (*n* = 2). Eighty patients completed the study. Of these, six were excluded from our analyses for having inflammatory conditions requiring vitrectomy, such as endophthalmitis and retained lens fragments following cataract surgery, leaving 74 subjects in our final data analysis. There were 51 males and 23 females in the study. There were a greater number of Black patients than any other race category (Table 2) overall. Thirty-five patients had PDR (case group) and 39 did not have PDR (control group). Of the 39 in the control group without PDR, 31 patients were nondiabetic while 8 were diabetic patients who had either no retinopathy or retinopathy that was less than proliferative.

Table 3 lists selected cytokines that have significant associations with PDR in our study, as well as those that have been shown in previous studies to be associated with diabetic retinopathy [10,11,12,13,14,15,16,17]. In our primary analysis, after accounting for *p*-values and FDR, we found significant upregulation of IL-8, IL-16, VEGF and VEGF-D in those with PDR compared to those without PDR. For our secondary analysis (adjusted for age, sex, and additional covariates of nonproliferative retinopathy, anti-VEGF therapy, and RRD), the same cytokines maintained and strengthened their significance with increased effect sizes, with additional cytokines IL-15, ICAM-1 and VCAM1 meeting the significance threshold. Effect size for cytokines with statistically significant elevations were almost twice as high in our secondary analysis compared to our primary analysis with similar *p*-values among cytokines in both analyses. All cytokines showing significant association with PDR were noted to have an increased fold change with the exception of bFBF, which was found to be lower in our case groups compared to our controls. Validation tests (VIF and R^2^) of the regression model showed no evidence of multicollinearity (see Table S1, Supplementary Materials).

## 4. Discussion

Our study is the first, to our knowledge, to identify novel increases of IL-15, IL-16, as well as decreased levels of bFGF in the vitreous of PDR patients. Additionally, our outcomes are in line with those of prior studies confirming increased levels of VEGF, ICAM-1, VCAM-1 and SAA as well as IL-8 and CRP, all of which have previously been found to be altered in the vitreous of PDR patients (Table 4) [11,12,13,14,15,16,17]. Most cytokines showed increased significance with greater effect sizes after adjusting for the presence of nonproliferative retinopathy, treatment with anti-VEGF therapy, and RRD, with the exception of bFGF.

Elevated levels of IL-15 and IL-16 in our study indicate that these biomarkers may play a significant role in the pathogenesis of PDR. While Hang et al. identified elevated levels of IL-15 in the serum among type 2 diabetics, this has not been previously identified in the vitreous humor [18]. Sato et al. identified elevated levels of IL-15 in the vitreous of active retinopathy of prematurity (ROP), which suggests that IL-15 may be upregulated in ischemic conditions such as diabetes and ROP that lead to angiogenesis [19]. IL-15,a proinflammatory cytokine that promotes activation of T-cells, neutrophils, and macrophages, is expressed in several inflammatory disorders, including rheumatoid arthritis, psoriasis, and autoimmune diabetes [19]. Similarly, IL-16 is a lymphocyte chemoattractant factor and stimulates lymphocytes, monocytes, and eosinophils [20]. Based on their known functions, we speculate that these cytokines contribute to leukostasis and microvascular damage. Leukocytes that are bound to the surface of capillary endothelial cells may release additional local cytokines or physically occlude the vessels, resulting in local ischemia downstream to the blockage [21]. Studies have shown elevated numbers of intravascular polymorphonuclear leukocytes adjacent to areas of capillary nonperfusion in diabetic monkeys as well as leukocytes accumulated in choroidal vessels of humans with diabetes [22,23].

Our study also reveals significant decrease levels of bFGF in the vitreous of PDR patients, but it loses its significance after adjusting for the presence of nonproliferative retinopathy, anti-VEGF treatment therapy, and RRD. This is in conflict with what has been reported in prior studies, which have demonstrated that bFGF levels are increased in patients with PDR [24]. As a growth factor, bFGF fosters fibroblast growth and proliferation leading to angiogenesis and tissue repair [25,26]. The mechanism has been shown to be through the ERK1/2 MPAK pathway leading to VEGF-A expression [25]. Previously, it has been shown that bFGF is elevated in the presence of proliferative vitreoretinopathy (PVR), a complication of retinal detachments [24]. Our findings with regard to bFGF, in consideration with prior studies, warrant further investigation.

Patients with rhegmatogenous retinal detachments (RRD) have been shown in prior studies to have a distinct cytokine profile. Adjusting for the presence of RRD in our study did not change the cytokine effect direction (Table 3) and the significant increases for IL-8, IL-15, IL-16, VEGF D, ICAM-1, and VCAM-1 remained with improved effect sizes. However, the significance for IL-17A, bFGF, VEGF-C, and SAA were lost with a reduction in effect sizes, indicating that the presence of RRD may have confounded the primary results for these 4 inflammatory markers. Prior studies on the cytokine profiles for vitreous samples from patients with RRD are limited. Takahashi et al. compared the cytokine profiles between patients with RRD and controls (MH, ERM, PDR and retinal vein occlusion). They did not find a statistically significant increase in IL-17A levels among those with RRD [27]. They also noted that VEGF levels were significantly higher in PDR controls than patients with RRD [27]. Interestingly, they noted that bFGF demonstrated a decrease in the PDR group compared to RRD, though this was not statistically significant. Other studies have also noted an increase in IL-6 and IL-8 in the vitreous of patients with RRDs compared to controls [28,29,30,31]. IL-16 was noted by Balough et al. to be elevated in vitreous of RRD patients compared to controls (those with ERM) [28]. Since patients with RRD were in the control group in our study, we believe the significant association of IL-16 to PDR is a true association, as increased IL-16 in patients with RRD would have biased towards the null hypothesis. Furthermore, the association between IL-16 and PDR remained when adjusting for RRD patients.

Several previously reported vitreous markers for PDR, including TNF-alpha, IL-6, IL-10, IL-13, MIP-1β, INF-γ and MCP-1, were not found to be elevated in our study [8,11,14,16,32,33]; this is consistent across both of our analyses. Future studies with more patients will be necessary to determine the importance of these cytokines in PDR. A summary of similar previous studies is included in Table 4.

A strength of our study lies in being able to clinically isolate patients with PDR and compare them to vitreous samples from those without PDR, and subsequently test if the significant associations remained after adjusting for other potential variables. Additionally, we were able to conduct this study in an urban academic medical center on a racially diverse group of patients, making our results more broadly generalizable.

Our study had limitations. Any external or pathological alteration of ocular physiology, such as injection of anti-VEGF medications, may confound analyses of vitreous biomarkers in a diabetic state. Some patients in our control and study groups had various and occasionally multiple ocular diagnoses at the time of the vitrectomy, and the presence of these additional ocular comorbidities could alter the inflammatory cytokine profile we obtained in our vitreous samples. Additionally, as diabetic retinopathy is graded on a continuous scale rather than absolute categories, the cytokine profiles obtained from our subjects with milder stage of diabetic retinopathy may likewise reflect values over a spectrum rather than definitive values representative of each class of nonproliferative diabetic retinopathy (mild, moderate and severe). We attempted to mitigate for some of these limitations by rerunning the regression analysis in an effort to reduce the effect of these variables. Furthermore, while adjusting for race is standard in clinical research, we decided against adjusting for race in our analysis due to the presence of several race categories and an imbalance of Black versus other races in the case and control groups. Black and White patients comprised 48% and 23% of total cases with PDR, respectively, and 26% and 44% of controls. This imbalance introduced bias in our statistical model and because of this we did not feel it appropriate to include race as a covariate. The higher proportion of Black patients in our case group with PDR versus control group is unsurprising as PDR is known to occur with higher incidence in Black individuals with diabetes [37].

## 5. Conclusions

Our findings identified novel increases in IL-15 and IL-16 among patients with PDR compared to controls. We also confirmed the elevation of several biomarkers (VEGF, ICAM-1, VCAM-1, SAA, IL-8 and CRP), as previously described in the vitreous of patients with PDR [9,10,11,12,13,14,15,16]. As a leading cause of blindness among adults in the US, diabetic retinopathy continues to present diagnostic and therapeutic challenges to clinicians. These findings warrant further investigation to determine if they are potential avenues for targeted therapies that can aid ophthalmologists in treating severe complications of diabetic retinopathy to salvage and improve vision.

## Figures and Tables

**Table 1:** Indications for surgery among patients with and without proliferative diabetic retinopathy.

With PDR	*n* = 35	%
Tractional Retinal Detachment	12	34
Vitreous Hemorrhage	18	51
Epiretinal Membrane	2	5
**Without PDR**	***n*** **=** **39**	**%**
Epiretinal membrane	3	8
Macular hole	5	13
Posterior vitreous detachment with vitreous hemorrhage	2	5
Rhegmatogenous retinal detachment	26	67
Retinal tear with hemorrhage	1	2.5
Subluxed lens	1	2.5
Vitreomacular traction	1	2.5

**Table 2:** Basic Demographics among Study Participants.

	PDR *n* = 35	%	Diabetics without PDR and Nondiabetics *n* = 39	%
**Gender**				
Male	23	65.7	28	71.8
Female	12	34.3	11	28.2
**Mean Age**	55.5		53.7	
**Race**				
White	8	23	17	44
Black	17	48	10	26
Asian	0	0	3	8
American Indian/ Alaskan Native	4	11	5	13
Other	6	17	4	10
**Diabetic Retinopathy Status**				
Nondiabetics	0	0	31	95
Diabetics with no retinopathy	0	0	6	
Mild	0	0	1	2.5
Moderate	0	0	1	2.5
Severe	0	0	0	0
Proliferative	35	100	0	0

Demographic breakdown of study patients including gender, race and diabetic retinopathy status.

**Table 3:** Regression Analysis for Vitreous Cytokines in Proliferative Diabetic Retinopathy (PDR).

		PDR Versus Non-PDR			PDR vs. Non-PDR, after Adjusting for Less Than Proliferative Diabetic Retinopathy, Anti-VEGF Therapy, and Rhegmatogenous Retinal Detachment		
		(*n* = 35 vs. 39)					
Cytokines	Fold Change	Effect (SE)	*p*-value	FDR	Effect (SE)	*p*-value	FDR
IL-8	1.24	1.03 (0.398)	**0.012**	**0.094**	2.09 (0.613)	**0.001**	**0.019**
IL-13	1.2	0.202 (0.123)	0.105	0.27	0.411 (0.194)	**0.038**	0.102
IL-15 *	1.2	0.448 (0.205)	**0.032**	0.131	0.891 (0.358)	**0.016**	**0.061**
IL-16 *	1.18	0.723 (0.289)	**0.015**	**0.094**	1.57 (0.506)	**0.003**	**0.028**
IL-17A	1.34	0.183 (0.0837)	**0.033**	0.131	0.275 (0.15)	0.072	0.157
VEGF	2.76	3.94 (0.61)	**<0.001**	**<0.001**	5.14 (1.07)	**<0.001**	**<0.001**
bFGF *	0.617	−1.67 (0.619)	**0.009**	0.094	−0.131 (0.961)	0.892	0.892
Flt1	1	0.0196 (0.355)	0.956	0.983	0.65 (0.507)	0.205	0.266
Tie2	1.54	1.63 (0.942)	0.088	0.263	1.93 (1.51)	0.205	0.266
VEGF-C	1.69	1.43 (0.866)	0.104	0.27	2.45 (1.34)	0.071	0.157
VEGF-D	1.83	3.02 (0.788)	**<0.001**	**0.005**	3.6 (1.26)	**0.006**	**0.035**
CRP	1.07	1.23 (0.679)	0.075	0.225	1.76 (1.08)	0.109	0.191
ICAM1	1.06	0.947 (0.413)	**0.025**	0.129	1.91 (0.624)	**0.003**	**0.028**
SAA	1.16	2.34 (1.15)	**0.045**	0.162	3.21 (1.83)	0.084	0.164
VCAM1	1.04	0.63 (0.586)	0.286	0.509	1.94 (0.856)	**0.027**	**0.0843**

Primary and secondary analyses of cytokine profiles in PDR against control groups. Cytokines listed have significant associations with PDR in our study and in previous studies. Blue-highlighted columns list cytokine values for PDR versus non-PDR patients while adjusting for age and sex as covariates. Green-highlighted columns indicate association after adjusting for age, sex, non-proliferative retinopathy, prior anti-VEGF therapy and rhegmatogenous retinal detachment. Positive value indicates positive association, and negative value indicates negative association. Fold change is the mean level of the cytokine in cases compared to controls. FDR, or false discovery rate, is shown for both analyses, with an a priori cut-off value of 0.10, or 10%. Bolded values indicate statistical significance or those that fall under the FDR cut off. * Indicates novel cytokine associations not previously reported. SE = Standard Error.

**Table 4:** Compilation of Cytokines Studies for Proliferative Diabetic Retinopathy.

Study	Sample Source	Methodology	Findings	Comments
Loporchio et al.	Vitreous	Cross-sectional Prospective Case (*n* = 35) vs. Control (*n* = 39)	IL 8 ↑	
			IL 15 ↑	
			IL 16 ↑	
			IL 17A↑	
			VEGF ↑, VEGF D ↑	
			bFGF ↓	
			SAA ↑, ICAM1 ↑	
Diabetes Research and Clinical Practice [11]	Vitreous and Serum	Case (*n* = 46) vs. Control (*n* = 15)	ICAM-1 ↑ (vitreous and serum)	Cytokines elevated in both vitreous and serum; E-selectin and VCAM-1 not statically significant in vitreous
			VCAM-1 ↑ (vitreous and serum)	
			E-Selectin ↑ (vitreous and serum)	
			vWF ↑ (vitreous and serum)	
Diabetes Care [15]	Vitreous and Serum	Case vs. control (*n* = 20 in both groups)	VCAM↑-1 (serum and vitreous) After correcting for total protein VCAM-1 was lower in PDR patients. VEGF was elevated in PDR patients vs. controls	Total protein concentration in vitreous hemorrhage patients was measured as a control. Only VCAM-1 elevation in vitreous was statistically significant.
Diabetes Care [34]	Plasma and Serum	Cross-sectional Prospective Case (*n* = 115) vs. control (*n* = 39)	PgE_2_ ↑ CRP ↑ IL-6 and PgE_1_ were the same among controls and cases.	Although nonvitreous samples tested, CRP elevated in case samples, similar to our findings of CRP in vitreous
Japan Journal of Ophthalmology [16]	Vitreous	Case (*n* = 76) vs. Control (*n* = 23)	IL-6 ↑	Also reviewed factors in comparison to CRVO
			IL-8 ↑	
			IL-10 ↑	
			IL-13 ↑	
			IP-10 ↑	
			MCP-1 ↑	
			MIP-1b ↑	
			PDGF ↑	
			VEGF ↑	
			Multivariate analysis showed IL-10 and IL-13 positively correlated to VEGF and that PDGF was inversely correlated to VEGF	
PLOS One [35]	Vitreous	Case vs. Control (*n* = 17 in both groups)	IL1-b ↑	Study group was both NPDR and PDR.
			INF-g ↑	
			VEGF-A ↑	
			PGF ↑	
			IL-2 (no change)	
			IL-4 (no change)	
			IL-13 ↓	
Investigative Ophthalmology and Visual Science [36]	Vitreous Aqueous Serum	Case vs. control (*n* = 7 controls and 17 cases)	IL-8 ↑ (aqueous and vitreous)	No difference in serum cytokines in cases vs. controls
			PlGF ↑ (aqueous and vitreous)	
			VEGFa ↑ (aqueous and vitreous)	

Summary of current studies examining vitreous and serum cytokine profiles in patients with PDR against control groups. Our study is included at the top of the table for comparison.

## Data Availability

The data presented in this study are available in Table 1, Table 2 and Table 3.

## Supplementary Materials

The following are available online at https://www.mdpi.com/article/10.3390/cells10051069/s1, Table S1: Variance inflation factor and Goodness-of-fit (R2) for.
