# Supplementary material for: Cytokine Levels in Human Vitreous in Proliferative Diabetic Retinopathy
Source: Cells. 2021 Apr 30;10(5):1069. doi: 10.3390/cells10051069 (PMC8147162; doi:10.3390/cells10051069)
Supplement: Supplementary file 1 [file cells-10-01069-s001.zip › cells-1135647-supplementary.pdf]

Supplementary Table: Variance inflation factor and Goodness-of-fit (R^2) for

**Dependent Variable (Cytokine)**	**VIF of the main independent variable (Disease status)**	**Goodness-of-fit (R^2)**
IL-8	2.503003	0.168
IL-13	2.503003	0.118
IL-15	3.20184	0.159
IL-16	3.20184	0.193
IL-17A	3.20184	0.111
VEGF	3.20184	0.465
bFGF	2.503003	0.192
Flt1	2.503003	0.23
Tie2	2.503003	0.0575
VEGF-C	2.503003	0.163
VEGF-D	2.503003	0.218
CRP	2.503003	0.111
ICAM1	2.503003	0.184
SAA	2.503003	0.104
VCAM1	2.503003	0.195

VIF: variance inflation factor; R2: percentage of variability of cytokine level explained by the linear model of the disease status after adjusting for covariates including age, sex, nonproliferative diabetic retinopathy, anti-VEFG treatment, and rhegmatogenous retinal detachments.
